# A Culturally Adapted SMS Text Messaging Intervention to Promote Antiretroviral Therapy Adherence Among African Americans: Protocol for a Single-Arm Trial

**DOI:** 10.2196/21592

**Published:** 2020-12-10

**Authors:** Maulika Kohli, Elizabeth C Pasipanodya, Jessica L Montoya, Maria Marquine, Martin Hoenigl, Vanessa Serrano, Clint Cushman, Rogelio Garcia, John Kua, Verna Gant, Sarah Rojas, David J Moore

**Affiliations:** 1 HIV Neurobehavioral Research Program, University of California San Diego San Diego, CA United States; 2 San Diego State University/University of California San Diego Joint Doctoral Program in Clinical Psychology San Diego, CA United States; 3 Rehabilitation Research Center, Santa Clara Valley Medical Center San Jose, CA United States; 4 University of California San Diego San Diego, CA United States; 5 San Diego State University San Diego, CA United States; 6 Family Health Centers of San Diego San Diego, CA United States

**Keywords:** medication adherence, behavior modification, short message service, mHealth, HIV/AIDS

## Abstract

**Background:**

African Americans are disproportionally affected by HIV and have poorer rates of antiretroviral therapy (ART) adherence compared to other racial or ethnic groups in the United States. Factors associated with poor HIV disease outcomes are commonly associated with sociostructural barriers that prevent engagement with and retention in HIV care. SMS text messaging interventions to promote ART adherence among predominantly non-Hispanic White persons with HIV (PWH) have been shown to be efficacious; however, limited research has been devoted to culturally tailoring interventions for underrepresented racial/ethnic groups. Considering African Americans show poorer engagement along the HIV care continuum, we developed an individualized and culturally tailored two-way SMS text messaging intervention to improve ART adherence and associated virologic suppression among African American PWH.

**Objective:**

In this paper we describe the protocol of a culturally tailored individualized Texting for Adherence Building (iTAB) intervention in a 24- to 48-week, single-arm study.

**Methods:**

We developed a culturally tailored iTAB intervention, which we are implementing in a 24- to 48-week, single-arm study. Participants were recruited from the Family Health Centers of San Diego (FHCSD), a federally qualified health center. Patient inclusion criteria were (1) receiving care at the FHCSD, (2) living with HIV, (3) self-identification as Black, African American, or of African ancestry, (4) English speaking, (5) age 18 or older, (6) currently on ART, and (7) able to provide informed consent. Study enrollment began in November 2017 and closed in July 2019. A total of 90 participants from the FHCSD enrolled in the iTAB intervention, and we anticipate completing data collection in July 2020. Participants were assisted in individualizing and customizing their SMS text message preferences at the baseline study visit. Self-assessment measures are collected at baseline, interim, and final study visits. Problems related to sending/receiving SMS text messages and barriers to ART adherence are assessed at each interim study visit. The FHCSD staff monitors and tracks participants’ daily SMS text message responses to ART adherence reminders using a clinical dashboard.

**Results:**

We hypothesize that the proportion of individuals achieving HIV virologic suppression (viral load <40 copies/mL) will be greater at the end of the intervention period compared to the proportion prior to study implementation. Additionally, we anticipate that rates of virologic suppression at the end of the intervention among participants receiving iTAB will be comparable to those among the general FHCSD non-African American population who did not receive iTAB. Finally, we anticipate a high response rate to iTAB SMS text messages as well as positive participant feedback at the end of the intervention with regard to the acceptability of, satisfaction with, and perceived efficacy of iTAB.

**Conclusions:**

The iTAB intervention is a novel individualized two-way SMS text messaging intervention that has been culturally tailored for use among African Americans with HIV. We anticipate that iTAB will demonstrate efficacy in future randomized control trials and will be supportive of medication adherence among other populations facing health disparities.

**International Registered Report Identifier (IRRID):**

DERR1-10.2196/21592

## Introduction

Across the United States, African Americans are disproportionally affected by HIV compared to other racial or ethnic groups, accounting for approximately 42% of persons with HIV (PWH) [1,2]. As national rates of HIV diagnosis are declining, incident rates among African Americans remain the highest among racial/ethnic groups [3]. In 2017, African Americans accounted for 42% of new HIV diagnoses in the United States, nearly 8 times the rate of infection among non-Hispanic Whites [2]. African Americans have poorer outcomes along the HIV care continuum, including decreased retention in care, lower likelihood of having an antiretroviral therapy (ART) prescription, worse ART adherence, and lower likelihood of achieving viral suppression [4-6]. These disparities along the HIV care continuum significantly contribute to worse overall health, risk of early morbidity, and heighted HIV-related burden compared to other racial/ethnic groups. Indeed, African Americans account for approximately 52% of those who have died from HIV [1]. Thus, providing support for sustained engagement in HIV care among African American PWH is a public health priority.

Factors associated with racial and ethnic disparities in rates of ART adherence influence adherence multidimensionally, operating at the structural (eg, political climate), health system (eg, accessibility of resources), community (eg, societal norms), interpersonal (eg, social support), and individual levels (eg, individual beliefs) [7,8]. Poor health literacy, dissatisfaction with health care providers, insufficient access to mental health services, fear or experience of HIV-associated stigma, incarceration, and poverty pose significant barriers to maintaining optimal ART adherence among African Americans living with HIV [8-12]. Despite efforts to provide widespread access and promote engagement in HIV care [13], many socio-structural barriers that disproportionally affect African Americans living with HIV continue to limit their utilization of health care services [14]. Therefore, developing culturally tailored ART adherence interventions are essential to improving HIV-related health outcomes [15], thereby reducing racial/ethnic health disparities among PWH.

Mobile health-related technologies have the potential to improve access to health care and promote health care management among a broad range of patients, including individuals from racial/ethnic minority groups [16]. Mobile phone ownership is now nearly universal, with 96% of adults in the United States owning a cellphone (including 98% of adult African Americans) in 2019 [17]. SMS text messaging is the most common and frequently used function of mobile phones, comprising 92%-100% of mobile phone usage among adults over the course of a typical week in the United States [18]. Given the ubiquity of mobile phones and high usage of SMS text messaging services, interventions delivered via SMS text message have the potential to support engagement in health care and in health behaviors.

In addition to being low-cost scalable alternatives to in-person interventions, SMS text messaging interventions can be efficacious [19-21]. For instance, the individualized Texting for Adherence Building (iTAB) intervention is a bidirectional SMS text messaging system that was designed to deliver automatic and individualized SMS text messages to assess and promote medication adherence among PWH. The efficacy of iTAB to improve adherence and dose timing has been evaluated among various populations at-risk for lower adherence (eg, ART adherence among PWH with co-occurring methamphetamine use disorder and co-occurring bipolar disorder) [22-24]. Despite evidence of efficacy among individuals from these vulnerable populations, previous intervention studies implementing bidirectional SMS text messaging medication reminders, including iterations of iTAB and other SMS text messaging interventions, may lack sensitivity toward the specific factors affecting ART adherence among African Americans living with HIV [25].

Our research team from the University of California, San Diego (UCSD) HIV Neurobehavioral Research Program (HNRP) and Antiviral Research Center (AVRC) has partnered with one of the top 10 largest (based on the number of patients served) federally qualified health centers, the Family Health Centers of San Diego (FHCSD), to meet the unique sociocultural needs of African American PWH in San Diego by adapting the iTAB intervention. FHCSD is the largest comprehensive HIV/AIDS services provider in the San Diego region, and 11.3% of its patient population are African American. When this project was conceived in 2016, only 77% of African Americans receiving care at FHCSD were virologically suppressed as compared to 90% of the non-African American clinic population. Given this significant disparity, we aimed to implement a culturally tailored bidirectional SMS text messaging intervention to improve ART adherence and related HIV disease outcomes among African Americans receiving care at FHCSD.

This study is a longitudinal project (Individual Community Care for HIV/AIDS Now: Getting Engaged—iC-CHANGE) funded by the California HIV Research Program (CHRP: HD15-SD-059). The formative phase of our intervention study assessed supports and barriers to ART adherence and solicited opinions among a sample of African American FHCSD patients and their providers of HIV care on how to best tailor the iTAB intervention. Suggestions for modification of the iTAB SMS text messaging system included: (1) matching the degree to which SMS text messages disclose HIV-related content depending on participants’ level of comfort; (2) variability of SMS text messages, incorporating positive, inspirational, entertaining, and culturally tailored content to their health; (3) personalization of SMS text message content and timing of SMS text messages; (4) positively reinforcing SMS text messages to encourage future adherence; and (5) tracking adherence over time using calendars to facilitate conversations between patients and care coordinators [26]. We also included culturally relevant health messages and trivia factoids (ie, messages including facts and quotes from historical African American figures and trivia pertaining to African Americans, Africa, or the African diaspora) to the extant pool of SMS text messages. Altogether, these features were incorporated into the current iTAB intervention to meet the diverse needs of the target population.

In this paper we describe the protocol followed in the implementation of the tailored iTAB intervention in a 24- to 48-week, single-arm study to evaluate changes in ART adherence and associated virologic suppression among African American FHCSD patients. Early formative work for this study with FHCSD revealed that providing participant’s the choice of the length of the intervention (ie, either 24 or 48 weeks) would encourage participation. We hypothesize that a greater proportion of participants will be virologically suppressed (viral load <40 copies/mL) at the end of the intervention period (week 24 or week 48) compared to the proportion of individuals suppressed prior to study participation. Additionally, we hypothesize that rates of virologic suppression at the end of the intervention among participants receiving iTAB will be comparable to rates among the general FHCSD non-African American population who did not receive iTAB. Finally, we anticipate a high response rate to iTAB SMS text messages as well as positive participant feedback at the end of the intervention with regard to the acceptability of, satisfaction with, and perceived efficacy of iTAB.

As an exploratory analysis of service utilization, we will examine whether participation in the iTAB intervention increases engagement in health care and utilization of services at the FHCSD, including fewer missed appointments and greater use of mental health and substance use disorder services. If found to be efficacious among our sample of African Americans living with HIV, the modified iTAB intervention could be used in other settings serving PWH to address, in part, the growing disparity in HIV treatment and care across the United States.

## Methods

### Participants, Interventions, and Outcomes

#### Study Setting

Participants were recruited from the FHCSD, a community health organization in Southern California dedicated to providing primary and mental health care to underserved diverse populations. FHCSD is the largest provider of HIV services in the San Diego area and receives Ryan White Part C funds. During the study, participants continue receiving health services at FHCSD and will also attend study visits at FHCSD. Participant data are stored and managed at the UCSD HNRP Center and AVRC.

Study visits are facilitated by care coordinators at the FHCSD. Care coordinators at the FHCSD are required to successfully complete trainings in clinical practices related to research with human participants and HIV education. The investigative team at UCSD’s HNRP and AVRC is required to successfully complete training according to standards of the Health Insurance Portability and Accountability Act (HIPAA) and National Institutes of Health, including annual training in “Research Aspects of HIPAA” and “Principles of Human Research Subjects Protection,” which emphasize the privacy rights of patients and research study participants and confidentiality safeguards for protected health information.

#### Eligibility Criteria

Patient inclusion criteria were (1) receiving care at the FHCSD, (2) living with HIV, (3) self-identification as Black, African American, or of African ancestry, (4) English-speaking, (5) age 18 or older, (6) currently on antiretroviral treatment, and (7) able to provide informed consent. Exclusion criteria were minimal, including visible intoxication at the time of the first study visit, having a diminished capacity to provide consent (eg, due to severe neurologic disease, active psychosis, or diminished capacity), or receiving hospice care.

#### Sample Size

Study enrollment began in November 2017 and closed on July 31, 2019. A total of 90 participants from the FHCSD enrolled in the iTAB intervention (ie, 90% of planned enrollment). Given the timing of enrollment of the last study participant, we anticipate to complete data collection in July 2020. Thus far, we have had 2 participants voluntarily withdraw from the study.

#### Recruitment

Potential participants were identified by FHCSD staff and invited for study participation through a variety of ways. In particular, the FHCSD care coordinator briefly reviewed the medical records of individuals with upcoming scheduled appointments in order to approach potentially eligible individuals at their in-person clinic visits. Potentially eligible individuals without upcoming appointments were also identified from their medical records and invited by phone to participate. Additionally, potentially eligible individuals were referred to the care coordinator by clinical care providers or self-referred themselves into the study after responding to flyers and recruitment cards. The care coordinator provided a thorough explanation of the study aims and participant responsibilities (eg, commitment to study visits and SMS text message responding) at the time of recruitment.

#### Participant Timeline

**Figure 1:**
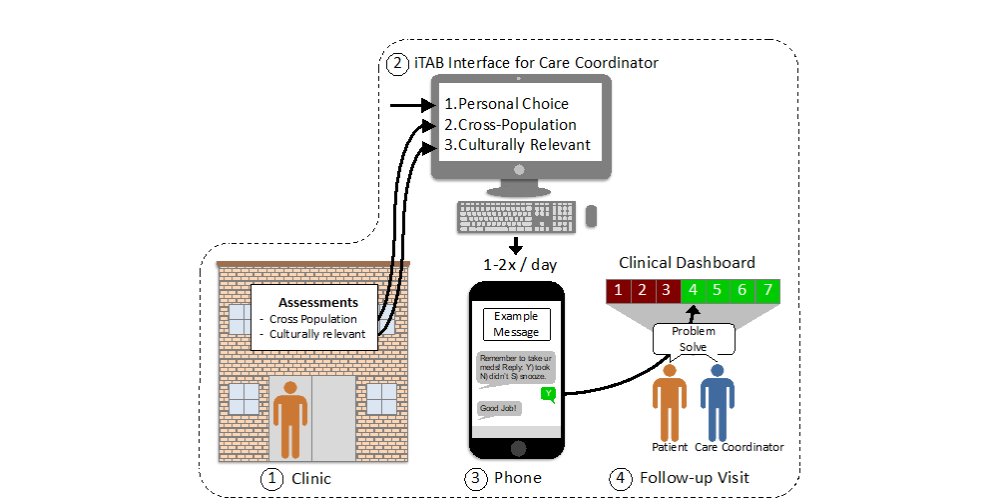
Schematic depicting study timeline, including iTAB setup. iTAB: individualized Texting for Adherence Building.

Participants receive the culturally tailored iTAB intervention, consisting of personalized SMS text messages sent daily to promote ART adherence. Participants have the option to enroll in a 24-week study with 3 visits (baseline, Week 12, and Week 24) or a 48-week study with 5 visits (baseline, Week 12, Week 24, Week 36, and Week 48). The initial visit lasts up to 3 hours and consists of completing a comprehensive battery of psychosocial assessments and setting up the iTAB intervention (eg, customizing SMS text message content and delivery time). Participants return every 12 weeks for interim visits, where they complete psychosocial assessments and discuss any concerns regarding the iTAB system with the FHCSD staff. The final study visit, at either week 24 or 48, lasts approximately 1 hour and includes completing psychosocial assessments and completing an exit interview during which participants are asked to provide feedback on the intervention (see Figure 1 for a schematic depicting the participant timeline).

#### iTAB Setup

At the initial study visit, each participant meets with an FHCSD care coordinator to review the informed consent documents for the study, completes psychosocial assessments, and sets up the iTAB system to receive SMS text messages on their mobile phone.

During iTAB set up, the HIV care coordinator first discusses with participants the time of day they want to receive the daily medication reminder SMS text messages. Participants are encouraged to set their SMS text message delivery time to coincide with their regular ART administration time and are able to select different SMS text message delivery times for week days versus weekend days. Participants are also informed that the iTAB system automatically sends their daily SMS text messages at random times within a half-hour window of their selected target time to limit habituation.

With regard to customizing SMS text message content, participants can select between receiving “low-disclosure” SMS text messages (ie, SMS text messages with no mention of HIV or the presence of a health condition) or “moderate-disclosure” SMS text messages (ie, SMS text messages that might allude to the presence of a health condition or contain factoids related to HIV, but do not disclose HIV status). In order to protect against unintentional divulgence of sensitive health information, when participants elect to receive moderate-disclosure SMS text messages, they are asked to also provide a word or brief phrase by which their ART will be referred to within the body of the SMS text messages (eg, “For some, taking all of their medications is part of a healthy and happy life. Please take your [insert word or brief phase]).

Within each disclosure category (ie, low versus moderate), participants are instructed to select at least five and up to 14 health promotion domains (eg, social support, celebrate health, self-esteem) and at least five and up to 18 factoid domains (eg, music, sports, food) for SMS text message content variability. For illustration, a low-disclosure health promotion SMS text message within the social support domain reads, “You are an asset to your community!” Examples of SMS text messages within the health promotion and factoid domains for both low- and moderate-disclosure categories are listed in Tables 1 and 2. As shown in Table 1, domains and SMS text messages within each disclosure level are overlapping and similar. In addition to daily medication reminder SMS text messages, participants are scheduled to receive health promotion SMS text messages during the standard working week and factoid SMS text messages on weekend days.

**Table 1:** Examples of health promotion SMS text messages by disclosure level (low versus moderate).

Health promotion domains	Low-disclosure sample SMS text message	Moderate-disclosure sample SMS text message^a^
Celebrate health	This is a friendly reminder to help keep you feeling good.	To help keep you feeling good, this is a friendly reminder to take your *iC-CHANGE*
Time/Focus	It only takes a second!	It only takes a second to be adherent!
Social support	You are an asset to your community!	You are an asset to your community! Please take your *iC-CHANGE*
Family support	You have wonderful family and friends.	Your family wants you to be happy and healthy! Please take your *iC-CHANGE*
Prevent disease	Take charge of your health!	Protect your health. Please take your *iC-CHANGE*
Self-esteem	You are special.	You are special. Please take your *iC-CHANGE*
Believing in yourself	You’ve been doing great!	You’ve been doing great with your adherence!
Dangers of poor habits/nonadherence	Being healthy won't happen if you don’t work for it.	Meds don’t work if you don’t take them. Please take your *iC-CHANGE*
Religious (Christian)	We need God as much in the calm as in the storm—Jack Hyles	We need God as much in the calm as in the storm—Jack Hyles
Religious (non-Christian)	Nothing is ever lost in following one’s own dharma—Bhagavad Gita	Nothing is ever lost in following one’s own dharma—Bhagavad Gita
Linking meds to behaviors	For some people, health behaviors are easier done soon after they wake up.	For some, taking meds is made easier when paired with something they do every day.
Other med taking	Remember that it helps you in different ways to stay healthy and happy.	Remember that all of your medications help you in different ways to stay healthy and happy. Please take your *iC-CHANGE*
SMS text messages from your provider	Wellness is key to a healthy life.	Wellness is key to a healthy life.
FHCSD^b^ support SMS text messages	FHCSD offers a wide range of health care services, including dental clinics, mental health, and substance abuse treatment.	FHCSD offers a wide range of health care services, including dental clinics, mental health, and substance abuse treatment.

^a^In the moderate-disclosure SMS text messages, participants were asked to provide a word or brief phase by which their medication would be referred to within the body of the text SMS text messages (ie, instances of *iC-CHANGE* in the moderate-disclosure SMS text messages was a space-filler and indicated whether the word or brief phase would be placed in the SMS text message).

^b^FHCSD: Family Health Centers of San Diego.

**Table 2:** Examples of factoid SMS text messages.

Factoid domains	Sample SMS text message
Health facts^a^	Our eyes never grow, but our nose and ears never stop growing.
HIV facts^b^	About 1/4 of HIV+ people have hepatitis C.
Affirmations	My body is healthy; my mind is brilliant; my soul is tranquil.
Fashion	The skirt is the 2nd oldest piece of clothing, outdated only by the loincloth.
Food	An ounce of chocolate contains about 20 mg of caffeine.
History	Madame Walker invented hair straightening formula and became the first female African American millionaire.
Jokes	What's the best thing about Switzerland? Not sure, but the flag is a big plus.
Lifehack	Can't sleep? Inhale for 4 seconds. Exhale for 8 seconds. Repeat. This will relax your body right to sleep.
Music	Michael Jackson bought the rights to most of the Beatles' music for US$47.5M in 1985. It's now worth US$450M.
Quotes	Seven days without laughter makes one weak. -Mort Walker
Science	The hottest planet in the solar system is Venus, with an estimated surface temperature of 864 F (462 C).
Southern California	The Ruins of Bombay is a town on the Salton Sea, one of the lowest settlements in altitude in North America.
Sports	Pittsburgh is the only city where all the major sports teams (MLB, NHL, NFL) have the same colors: Black/gold.
General trivia	The word malaria means bad air. This derives from when it was thought that diseases were caused by dirty air.
TV/Movies	The 1st movie with sound was The Jazz Singer (1927). 1st words: 'Wait a minute, you ain't heard nothing yet'.
Word of the Day	kalimba: a plucked instrument of African origin.
SMS text messages about health^a^	Take care of your body. It's the only place you have to live.—Jim Rohn.
SMS text messages about HIV^b^	HIV changed my life, but it doesn’t keep me from living.—Magic Johnson

^a^Factoid domain present only in the low-disclosure setting.

^b^Factoid domain present only in the moderate-disclosure setting.

After customizing SMS text message content, participants have the option to personalize their response options to the iTAB SMS text messages. The standard iTAB setup provides participants with 3 response options to the iTAB SMS text messages: Y) Yes, N) No, and S) Snooze. However, participants are able to select alternative response options that maintain the same letter codes: for example, Y) Yeh, N) Nah, and S) Zzzz.... Participants are also asked to select at least ten positive reinforcement SMS text messages from a pool of 26 SMS text messages. Reinforcement SMS text messages are sent to participants after they send an SMS text message response indicating ART adherence. A typical reinforcement SMS text message reads, “Great job!” Participants are also able to customize the reinforcement SMS text messages by composing their own reinforcers.

**Figure 2:**
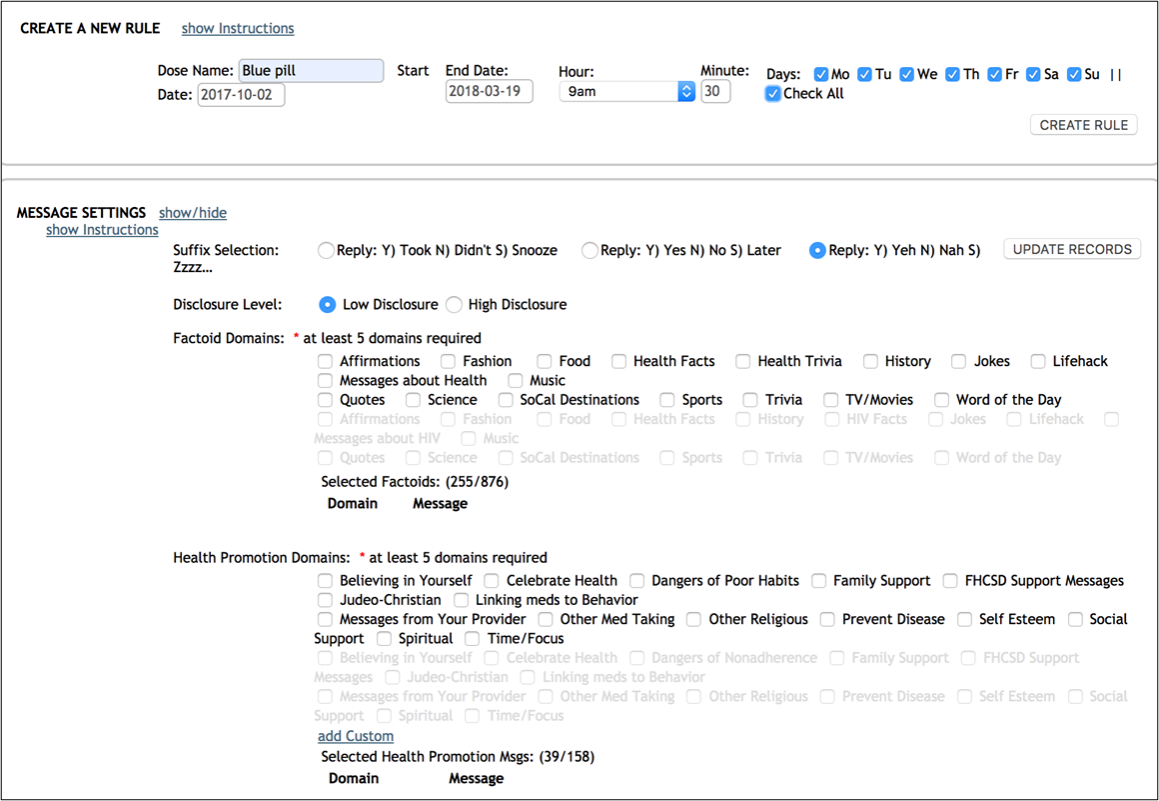
iTAB interface with SMS text message selection options. iTAB: individualized Texting for Adherence Building.

**Figure 3:**
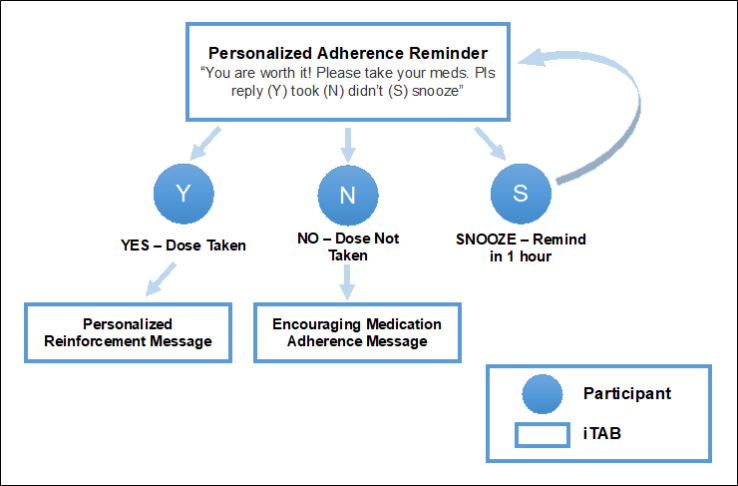
iTAB SMS text message decision system. iTAB: individualized Texting for Adherence Building.

Once participants make their various SMS text message selections, the care coordinator reviews the settings with the participant before they are saved to the secure iTAB system database (Figure 2 shows a screenshot of the iTAB interface with SMS text message selections). Prior to concluding the baseline visit, a sample SMS text message is sent to ensure the participant can receive SMS text messages and successfully respond to the system. Once the iTAB system is set up for an individual, automatically generated SMS text messages can be sent to the participant following a decision tree algorithm (Figure 3). For example, a personalized reminder SMS text message might read, “Be covered! It’s dose time.” If the participant replies “Y,” a personalized positive reinforcement SMS text message is automatically sent to the participant (eg, “Keep up the good work!”). If a participant replies “N,” an encouraging SMS text message to take their medications is sent (eg, “Please take a moment for your health ASAP. Call us if you need assistance or have a question”). If a participant responds “S,” a follow-up reminder SMS text message is sent after an hour delay (eg, “Hi. You did not take a break for yourself an hour ago, have you been able to do so since then?”). Finally, if a participant sends an unexpected response to an SMS text message (ie, anything other than a “Y”, “N”, or “S”), the iTAB system sends an automatic response to the participant stating that their response was not understood.

#### Participant Monitoring

Within 2 weeks of the baseline visit, participants are contacted by an FHCSD care coordinator over the phone to discuss any issues related to receiving and responding to SMS text messages. Participants are also contacted by an FHCSD care coordinator 4 weeks prior to their in-person study visits to schedule their upcoming appointment. At interim study visits (Week 12, 24, and 36, depending on the duration of the intervention), participants are asked to complete a set of psychosocial questionnaires and discuss concerns regarding the iTAB system. Additionally, participants and care coordinators reassess SMS text message customizations at each interim clinic visit to ensure participant satisfaction with the system and to optimize ART adherence.

All SMS text messages sent (eg, reminders, reinforcement SMS text messages) and received (eg, participant responses) by the iTAB system are recorded. After 3 consecutive days of nonresponse to SMS text messages or “N” responses to prompts about medication adherence, an automated SMS text message regarding participant noncompliance to the study and to their medications is triggered. An FHCSD care coordinator attempts to contact the participant to discuss barriers to medication adherence (eg, forgetting dose time) and potential solutions (eg, pairing medication taking with another daily behavior, such as brushing one’s teeth).

**Figure 4:**
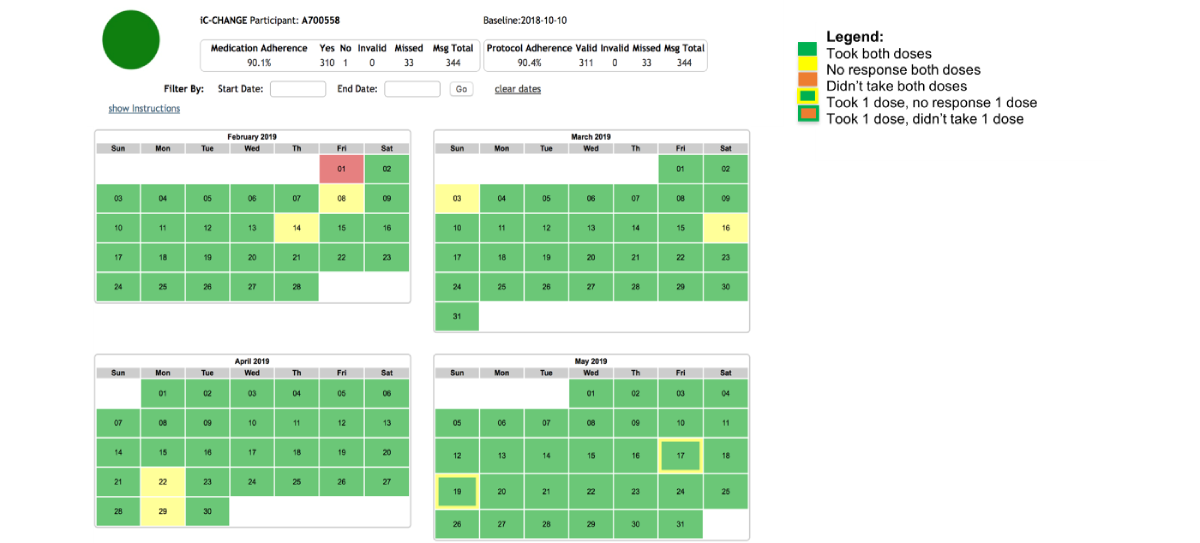
Example of the iTAB dashboard and summary of medication adherence with the Stop-Light System. Example of iTAB Calendar Report for ART adherence and SMS text message responses. These examples are of an individual on 2 doses of medication a day, receiving 2 iTAB SMS text messages per day. ART: antiretroviral therapy; iTAB: individualized Texting for Adherence Building.

The care coordinator is also able to monitor and track participants’ daily SMS text message responses to medication adherence reminders using the iTAB clinical dashboard. For ease of interpretation, the dashboard utilizes a “Stop-Light” system as a simple summary of the self-reported adherence rate (Figure 4). Adherence rates greater than 90% are displayed with a green circle, those between 70% and 90% with a yellow circle, and those less than or equal to 70% with a red circle. Furthermore, a monthly calendar summarizes past daily adherence and is color coded according to participants’ daily self-reported adherence (Figure 4). The iTAB clinical dashboard is used to facilitate conversations between the participant and care coordinators during interim clinic visits about ART adherence and to problem solve instances of nonadherence or nonresponse to SMS text messages. Information from the clinical dashboard is not shared with health care providers.

#### Outcomes

Undetectable plasma viral load and ART adherence (as measured by participants’ responses to daily SMS text messages) are the primary outcomes for this single-arm trial. In addition, participants complete an in-person comprehensive battery of psychosocial assessments at the baseline, interim, and end-of-study visits (at either Week 24 or Week 48). Specific assessments administered at each study visit are listed in Table 3. Assessments with strong reliability and validity among HIV populations were chosen, where possible. Measures collected at the baseline study visit include plasma viral load (abstracted from FHCSD electronic medical records), medication adherence behaviors (eg, percentage of adherence in the past 30 days), psychosocial risk factors associated with poor HIV-related outcomes among PWH (eg, sociodemographic characteristics, substance use, and social support), and culturally specific risks for nonadherence among African Americans (eg, subjective experiences of discrimination, HIV-related stigma, and medical mistrust). Measures collected at interim study visits (ie, every 12 weeks) are a subset of the baseline measures (eg, perceived stress, alcohol use, substance use, and depressive symptoms). Upon completion of the intervention (at either week 24 or 48), participants complete a subset of psychosocial assessments, as well as a feedback questionnaire assessing their experience, satisfaction with, and perceived usefulness of the intervention. Laboratory data on HIV-disease characteristics (eg, plasma viral load and CD4 count) and utilization of FHCSD services are collected from electronic medical records by care coordinators at each in-person study visit.

**Table 3:** Assessments administered^a^

Domain		Assessments	Baseline	Week 12, 36	Week 24, 48
**HIV disease variables and antiretroviral therapy adherence**					
	Primary outcome: Viral load^b^	Data acquired from FHCSD^c^ blood draw at clinical visit	X		X
	CD4 count^b^	Data acquired from FHCSD blood draw at clinical visit	X		X
	Self-report of ART^d^ adherence	Ira Wilson Adherence Questionnaire (IWQU) [27]	X	X	X
**Factors that may impact antiretroviral therapy adherence**					
	Sociodemographic characteristics	Self-reported sex, gender, age, race, ethnicity, birth location, relationship status, HIV status disclosure, employment, income, housing stability, education	X		
	Depression symptoms	Beck Depression Inventory-II (BDI)	X	X	X
	Alcohol and illicit substance use	Alcohol Use Disorders Identification Test (AUDIT) and Drug Abuse Screening Test (DAST-10 Modified)	X	X	X
	Subjective social status	MacArthur Scale of Social Status Scale	X		
	Beliefs related to HIV medications	Beliefs Related to Medications Questionnaire	X		
	Supports and barriers to adherence	Supports and barriers to adherence	X		
	Self-efficacy for medications	Self-efficacy for managing medications and treatments	X	X	X
	Illicit substance use and sexual behaviors	Risk Questionnaire	X		X
	Collective self-esteem	Collective Self Esteem Scale	X		X
	Medical mistrust	Trust in the medical system; attitudes toward the health care	X		X
	Instrumental and social support	NIH Toolbox Emotional Support, Instrumental Support, and Loneliness scales	X		X
	Beliefs about HIV	Beliefs about HIV	X		X
	Subjective experiences of discrimination	Everyday Discrimination Scale; Multiple Discrimination Scale	X		X
	Quality of life	Medical Outcomes Study: 36-item Short Form Survey (MOS: SF-36)	X	X	X
	Personality	Ten-Item Personality Inventory	X		
	Perceptions of stress	Perceived Stress Scale	X	X	X
	Lifetime exposure to stress/adversity	Negative life events	X		
	Health care utilization	Utilization of care	X		X
	Health literacy	Short Assessment of Health Literacy-English (SAHL-E)	X		
	Stigma and psychosocial aspects of living with HIV	HIV Stigma Scale	X		
	Religiousness/Spirituality	Brief Multidimensional Measure of Religiousness and Spirituality	X		
	Cellphone Usage	Cellphone Usage Questionnaire	X		
	Medication-taking behavior^e^	Medication-Taking and Text-Responding Behavior Questionnaire			X
	Intervention feedback^e^	iC-CHANGE Feedback Questionnaire (Likert scales of feasibility, acceptability, and satisfaction)			X

^a^The X denotes those visits where the assessment will be administered.

^b^Data collected from electronic medical records from FHCSD clinical visit during the study period or provided by the participant.

^c^FHCSD: Family Health Centers of San Diego.

^d^ART: antiretroviral therapy.

^e^Administered only at the final visit.

#### Participant Compensation

In consultation with FHCSD, we determined the amount of funds that would be appropriate for compensation. Participant compensation was consistent with levels used in other studies at FHCSD. The goal was to provide at least US $13 per hour, inclusive of travel time. Participants are compensated US $50 for the baseline visit and each interim visit. Additionally, they receive US $70 for their final visit, at either Week 24 or Week 48. Consequently, participants stand to be compensated up to US $170 for the 24-week study, or US $270 for the 48-week study.

### Data Collection, Management, and Analysis

#### Data Collection Methods

The iTAB system records all SMS text messages that are sent (eg, reminders, reinforcement SMS text messages) and received (eg, participant responses). Indicators of medication adherence as well as adherence to the intervention are collected via the iTAB system.

Measures (data derived from the electronic medical record and psychosocial assessments) are collected at baseline, interim, and the final study visits. Assessments within a given domain were selected based on reliability and validity among African Americans living with HIV. The self-assessments administered at each study visit are listed in Table 3. Participants are allowed to continue study participation at any point, even after multiple missed in-person visits or periods of nonresponsiveness to SMS text messages. If a participant is lost to follow-up, the care coordinator will attempt to contact the participant by phone on a weekly basis for a 1-month period. If contact is not made within the 1-month period, the care coordinator will attempt to contact the participant by phone on a monthly basis. The care coordinator can additionally check if the participant has a primary care appointment scheduled. If successfully contacted, the care coordinator will assess the participant’s unique barriers and challenges to committed participation in the study and attempt to collaboratively find appropriate solutions (eg, if the participant no longer has a cell phone, a study phone may be provided [up to 1 replacement phone]). Participants expressing the desire to terminate their study participation are formally withdrawn from the study and their data excluded from analyses.

#### Data Management

FHCSD has implemented a HIPAA-compliant electronic health record system. This system has built-in access controls, such as passwords and PIN numbers, limited access to protected information, encrypted storage, and an audit trail, which tracks records accessed and by whom. Medical information (eg, HIV status, toxicology results, and medical history) gathered by the FHCSD care coordinators at each in-person visit is deidentified so that investigators at UCSD’s HNRP and AVRC cannot link the unique study IDs to identifying information, such as names or medical record numbers. Participants’ actual names, medical record numbers, and other identifying information remain with the FHCSD at all times.

Participant self-report psychosocial assessments are completed at the FHCSD. To promote data quality, care coordinators continuously assess for unusual patterns of responding (eg, providing the same response to all questions) and missing questions at each study visit. Physical copies of the self-report psychosocial assessments are regularly transferred to the HNRP in a secured case by at least two study personnel. Prior to data entry, questionnaires are double-scored and flagged for missing responses using the code “999.” Assessments are then entered into the secure HNRP database using the participant’s deidentified study ID.

### Outcomes

The primary outcomes of the intervention are undetectable plasma viral load and self-reported ART adherence, as measured by participants’ responses to daily SMS text messages. We hypothesize that a greater proportion of iTAB participants will be virologically suppressed (viral load <40 copies/mL) at the end of the study period (either week 24 or 48) compared to the proportion at study entry. Currently, 77% of African Americans living with HIV receiving services at the FHCSD are virally suppressed. We hypothesize an increase of 11% (ie, from 77% to 88%) in the proportion of participants with undetectable viral loads at study completion. We additionally hypothesize that the rates of viral load suppression after the intervention will be comparable to the general FHCSD non-African American population who did not complete the iTAB intervention. Lastly, we hypothesize high engagement with the iTAB system reflected by high response rates to iTAB SMS text messages and positive feedback regarding the acceptability, satisfaction with, and efficacy of iTAB.

In addition to these primary anticipated results, we hypothesize that participants involved in the iTAB intervention will utilize more comprehensive FHCSD services, have fewer missed appointments, and greater use of substance use and mental health services compared to those not in iTAB.

### Statistical Methods

We will characterize HIV disease characteristics (eg, CD4 count) and psychosocial characteristics of the study population using descriptive statistics. Linear regressions will be used to examine psychosocial factors associated with ART adherence. The number and proportion of virologically suppressed participants at baseline and at the final study visit will be compared. We will use McNemar test for paired proportions to compare virologic control within-subjects using data from the baseline and final study visits. Based on a 2-sided, 1-sample test for proportions to compare the viral suppression rate before and after the 48-week study, we have 74% power to detect an increase of 11% in suppression rate (ie, from the reference 77% to 88%) with N=88 participants and alpha =.05.

In secondary/sensitivity analyses, multivariable conditional logistic regression analysis will be performed to study the association between demographic factors and plasma viral load, adjusting for baseline demographic variables and clinical characteristics. Variables with some association to plasma viral load (*P*<.10) will be included as covariates. Data from participants will also be analyzed using methods appropriate for examining longitudinal data, for instance, linear mixed-effect modeling/multilevel modeling, latent growth curve modeling, and growth mixture modeling. These model types will allow for interpreting fixed effects (average group trajectory), random effects (individual variability), and heterogeneity in shape of longitudinal change. All tests of significance for secondary outcomes will be 2-sided; following standard conventions, a *P*-value of less than .05 will be considered as statistically significant. Appropriate nonparametric alternatives will be considered, if parametric assumptions are not appropriate for the data.

The secondary outcomes of the intervention will be responses from the various Likert-type assessments administered at each study visit (eg, medical mistrust, provider satisfaction, barriers to health care access, and alcohol or illicit substance use) and the iTAB feedback questionnaire. We will use regression-based models to examine the associations between these nonadherence risk factors and other study outcomes (eg, association between perceived efficacy of iTAB and virologic suppression).

### Monitoring

#### Harms

No significant harms associated with participation in the iTAB intervention are anticipated. Significant and severe problems, including those related to mood, substance use, or structural barriers to adherence, are closely monitored and addressed appropriately. At each study visit, care coordinators and study investigators review participants’ psychosocial assessments and appropriately consider referral to mental health services. If participants endorse thoughts of harm to self or others, care coordinators triage the participant to the FHCSD clinical team for further evaluation of risk and for appropriate coordination of care.

#### Auditing

FHCSD and HNRP staff including primary investigators and care coordinators are responsible for data quality assurance and monitoring of study visit completions.

### Ethics and Dissemination

#### Research Ethics Approval

The study protocol was approved by the Institutional Review Board at UCSD and all participants provided written informed consent to participate.

#### Protocol Amendments

All protocol amendments will be submitted for approval by the UCSD Institutional Review Board. Study funders and key staff will be informed of protocol amendments. Study participants will review and sign a revised consent form in the event that the consent form is changed.

#### Consent or Assent

At the initial study visit, the care coordinators thoroughly review the consent form with the participant, specifically noting the descriptions of study procedures and potential risks to participating. Any individual who was unable to adequately complete the consent form was not enrolled in the study. If a participant’s capacity to consent was in question, the participant was evaluated by a clinical psychologist or FHCSD health care provider who made the final determination of decisional capacity. This decision and a detailed explanation from the clinician/health care provider was documented.

#### Confidentiality

Efforts to maintain and protect patient privacy are of the utmost importance. Recruitment of participants, informed consent procedures, and study visits are conducted individually in a private medical examination room in order to maintain confidentiality. Additionally, to ensure confidentiality, data linking participant IDs to their nonidentifiable study ID are stored in a secure database at the FHCSD. Only the participant’s code number appears on all data collection forms at the HNRP and FHCSD. Identifiable protected health information, including participant study information and signed consents, is stored in locked file cabinets within secured rooms at FHCSD.

Physical files containing deidentified quantitative data (ie, self-report psychosocial assessments) are routinely transported from the FHCSD to the HNRP in a locked briefcase by 2 study personnel (EP and RG). Quantitative data are entered into a database at the HNRP, and all physical records are stored in a locked file room. The only protected health information that is obtained or stored on a multilevel hardware/software firewalled and encrypted UCSD network is a phone number, which is necessary to send the SMS text messages.

The study design minimizes risk associated with using mobile communication. At study enrollment, each participant’s mobile phone number is linked to an anonymous ID that is verified through a 2-step authentication process. Furthermore, SMS text messages that arrive on participants’ phones are sent from a generic UCSD phone number and do not include any participant identifying information (eg, participant name). All text SMS text message data are secured on a private UCSD network and can only be accessed via a virtual private network and secure password-protected website. These systems comply with HIPAA regulations for protection of person-identifiable health data over the internet. An HNRP Confidentiality Committee performs regular inspections to monitor procedures.

## Results

### Study Enrollment

Study enrollment began in November 2017 and closed in July 2019. A total of 90 participants from the FHCSD enrolled in the iTAB intervention. Given the timing of enrollment of the last study participant, we anticipate completing data collection in July 2020. As of April 2020, 64 participants have completed the study. Two participants have withdrawn from the study, resulting in the total sample of 88 participants.

### Participant Characteristics

Baseline demographic characteristics are presented in Table 4. The mean age of participants was 46.4 (SD 11.9) years. Participants primarily identified as male, Black/African American/African origin, and reported some college education. In terms of employment status, nearly two-thirds of the sample were either currently unemployed or currently on disability. With respect to HIV disclosure, a majority of participants have disclosed their HIV status, with approximately three-fourths confiding in their friends and family.

**Table 4:** Baseline demographic characteristics (N=88).

Descriptive		Value	
Age, mean (SD)		46.4 (11.9)	
Education, mean (SD)		14.7 (13.2)	
Sex assigned at birth (male), n (%)		76 (86)	
**Gender identity**			
	Men, n (%)	73 (83)	
	Women, n (%)	13 (15)	
	Transman/Trans Male/Transmasculine, n (%)	0 (0)	
	Transwoman/Trans Female/Transfeminine, n (%)	1 (1)	
	Genderqueer/Gender nonconforming, n (%)	1 (1)	
**Race**			
	Black/African American/African origin, n (%)	74 (84)	
	Black mixed-race identity^a^, n (%)	14 (16)	
	Spanish/Hispanic/Latino Origin, n (%)	9 (10)	
Born in United States (Yes), n (%)		79 (90)	
Born in California (Yes), n (%)		33 (38)	
**Disclosed HIV status (Yes), n (%)**		79 (90)	
	Disclosed to family, n (%)	64 (81)	
	Disclosed to friends, n (%)	70 (89)	
**Employment status**			
	Full-time employment, n (%)	18 (20)	
	Part-time employment, n (%)	13 (15)	
	Currently unemployed, n (%)	27 (31)	
	Currently on disability, n (%)	24 (27)	
	Other^b^, n (%)	6 (7)	

^a^Black mixed race identity refers to identifying as Black as well as other racial identities including American Indian or Alaska Native, Asian American or Asian Origin, Native Hawaiian or Pacific Islander, or White.

^b^Includes looking for a job, applying for supplemental security income (SSI), applying for disability, or retired.

## Discussion

### Protocol Summary

This longitudinal study, titled “Individual Community Care for HIV/AIDS Now: Getting Engaged (iC-CHANGE),” aims to improve ART adherence among African Americans living with HIV using a culturally adapted bidirectional SMS text messaging intervention. Previous studies have demonstrated the efficacy of 2-way SMS text messaging interventions in supporting ART adherence; however, these studies primarily enrolled non-Hispanic white PWH despite epidemiological evidence of greater HIV-related burden among African American PWH [25]. This protocol details the implementation of an intervention that was adapted using feedback from African American PWH and their providers of care [26]. We anticipate that the tailored iTAB intervention will significantly improve ART adherence among the targeted population and, consequently, could be implemented among other African American PWH across the state of California.

### Challenges

In formative study phases, issues arose related to integrating community-based participatory research with clinical practice. Focus group discussants suggested that the study facilitate interactions between patients and their providers of clinical care [26]. Thus, we aimed to provide study participants with the choice to share their iTAB adherence calendars with their providers to promote conversations around adherence. FHCSD has a proprietary medical record system and integrating our research findings with health care providers in a reliable and secure fashion proved to be a substantial barrier. Future implementation science studies will aim to strategically incorporate iTAB with electronic medical records where health care providers can access study-derived outcomes to enhance clinical care.

### Future Directions

Should findings for this study indicate this intervention is useful in improving ART adherence, future research may include conducting a randomized control trial of the iTAB intervention among African Americans living with HIV against a control group. Following evidence of efficacy in a randomized control trial, we may also conduct a larger-scale implementation study across multiple settings. Additionally, future work will examine the possible unidentified moderators of intervention effects including structural, health system, community, interpersonal, and individual facilitators or barriers to adherence that may supersede the beneficial potential of iTAB. Furthermore, there may be other subgroups of PWH at FHCSD (eg, other racial/ethnic minorities, persons with low socioeconomic status, or intravenous drug users) that would similarly benefit from individualized adherence SMS text messages and should be the focus of future efficacy studies. Finally, future work will expand on the identification of key predictors and SMS text message types for improving ART adherence. For instance, some constructs assessed may have good predictive value of future decline in viral load and may be effective tailoring variables for supporting adherence in subsequent interventions.

### Conclusions

In summary, given the integration of the feedback from key stakeholders in a previous study, we anticipate that the modified iTAB intervention has great potential for improving HIV disease outcomes, including medication adherence and virologic suppression among African American PWH.

## Abbreviations

ART: antiretroviral therapy
AVRC: Antiviral Research Center
FHCSD: Family Health Centers of San Diego
HIPAA: Health Insurance Portability and Accountability Act
HNRP: HIV Neurobehavioral Research Program
iTAB: individualized Texting for Adherence Building
PWH: persons with HIV
UCSD: University of California, San Diego
